# Supplementary material for: Stool Glycoproteomics Signatures of Pre-Cancerous Lesions and Colorectal Cancer
Source: Int J Mol Sci. 2024 Mar 27;25(7):3722. doi: 10.3390/ijms25073722 (PMC11012158; doi:10.3390/ijms25073722)
Supplement: Supplementary file 1 [file ijms-25-03722-s001.zip › Soares et al IJMC 2024_supporting information (2).pdf]

Stool Glycoproteomics Signatures of Pre-Cancerous Lesions and Colorectal Cancer

-Supporting Information-

Janine Soares [1, 2, 3, #] and Mariana Eiras [1, #], Dylan Ferreira [1, 2, 4, 5, 6], Daniela A. R. Santos [1, 2, 7], Marta Relvas-Santos [1, 2, 5, 6, 8], Beatriz Santos [1], Martina Gonçalves [1], Eduardo Ferreira [1], Renata Vieira [9], Luís Pedro Afonso [1, 9], Lúcio Lara Santos [1, 2, 10, 11, 12], Mário Dinis-Ribeiro [7, 13, 14], Luís Lima [1, $] and José Alexandre Ferreira [1, 2, 11, $] *

[1] Experimental Pathology and Therapeutics Group, Research Center of IPO Porto (CI-IPOP)/RISE@CI-IPOP (Health Research Network), Portuguese Oncology Institute of Porto (IPO Porto), Porto Comprehensive Cancer Center Raquel Seruca (Porto.CCC Raquel Seruca), 4200-072 Porto, Portugal; [2] Institute of Biomedical Sciences Abel Salazar (ICBAS), University of Porto, 4050-313 Porto, Portugal ; [3] REQUIMTE-LAQV, Department of Chemistry, University of Aveiro, 3810-193, Aveiro, Portugal; [4] Center for Applied Medical Research (Centro de Investigación Medica Aplicada, CIMA), University of Navarra, 31008 Pamplona, Navarra, Spain; [5] i3S – Instituto de Investigação e Inovação em Saúde, Universidade do Porto, 4200-135 Porto, Portugal; [6] INEB-Instituto Nacional de Engenharia Biomédica, Universidade do Porto, 4200-135 Porto, Portugal ; [7] Faculty of Medicine (FMUP), University of Porto, 4200-072, Porto, Portugal; [8] REQUIMTE-LAQV, Department of Chemistry and Biochemistry, Faculty of Sciences, University of Porto, 4169-007, Porto, Portugal; [9] Department of Pathology, Portuguese Oncology Institute of Porto, Porto, Portugal; [10] FF-I3ID, University Fernando Pessoa, 4249-004, Porto, Portugal; [11] GlycoMatters Biotech, 4500-162, Espinho, Portugal; [12] Department of Surgical oncology, Portuguese Oncology Institute of Porto (IPO-Porto), 4200-072, Porto, Portugal; [13] Precancerous Lesions and Early Cancer Management Group, Research Center of IPO Porto (CI-IPOP)/Rise@CI-IPOP (Health Research Group), Portuguese Institute of Oncology of Porto (IPO Porto)/Porto Comprehensive Cancer Center Raquel Seruca (Porto.CCC Raquel Seruca), Porto, Portugal; [14] Department of Gastroenterology, Portuguese Oncology Institute of Porto, Porto, Portugal

Corresponding author:
José Alexandre Ferreira (jose.a.ferreira@ipoporto.min-saude.pt)
Experimental Pathology and Therapeutics Group, Research Centre, Portuguese Oncology Institute of Porto, R. Dr. António Bernardino de Almeida 62, 4200-072 Porto, Portugal; Tel. +351 225084000 (ext. 5111).
Keywords: proteomics; glycoproteomics; colorectal cancer; stool

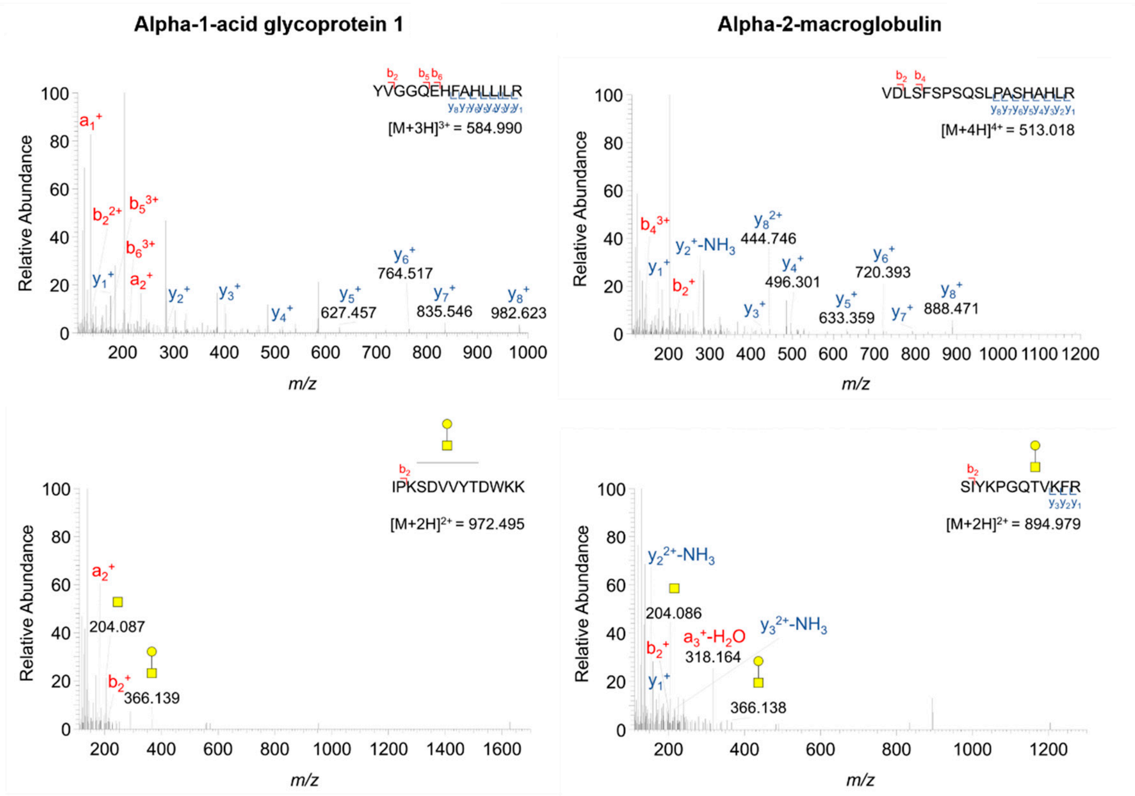

Supplementary Figure S1. MS/MS spectra for peptides not modified with glycosylation (top panel) and glycopeptides (bottom panel), supporting the expression of the glycoproteins alpha-1-acid glycoprotein 1 and alpha-2-macroglobulin in stool samples.

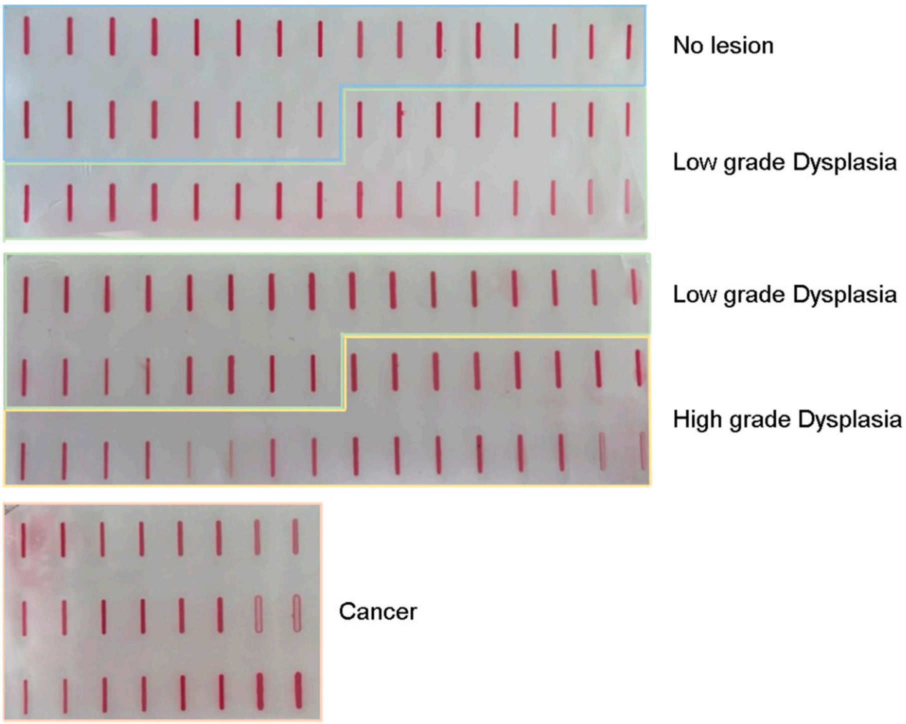

Supplementary Figure S2. Ponceau staining from stool sample' slot blot for T-antigen expression assertion.
